# Management of Difficult Airway in a Coronary Artery Bypass Graft Patient With Limited Oral Access

**DOI:** 10.7759/cureus.86504

**Published:** 2025-06-21

**Authors:** Avinash D'Souza, Priyanka Shenoy, Andrew Nolasco, Mark Schlesinger

**Affiliations:** 1 Anesthesiology, Hackensack University Medical Center, Hackensack, USA

**Keywords:** awake fiberoptic intubation, cardiac anesthesia, coronary artery bypass grafting (cabg), difficult airway management, mandible reconstruction

## Abstract

Management of difficult airways is a recognized challenge in anesthesia and can be further complicated by altered anatomy. We present a case of a 56-year-old male with a history of a mandibular jaw tumor resection requiring coronary artery bypass graft (CABG) surgery. The altered anatomy due to the patient’s jaw resection required awake intranasal fiberoptic intubation as there was limited oral access. Our discussion focuses specifically on adequate pre-induction preparation, hemodynamic monitoring, and pharmacological agents used for intubation. Despite the complexity of the case, our patient was successfully induced and intubated without any adverse outcomes. He ultimately underwent a two-vessel CABG with uneventful extubation.

## Introduction

Anesthesiologists utilize a variety of techniques in the perioperative setting to ensure safe airway management. The incidence of difficult airways, both predictable and unpredictable, during endotracheal intubation is approximated as between 0.3% to 13% [1]. The gold standard for managing predictable difficult airways is awake fiberoptic intubation. Awake fiberoptic nasal intubation requires the endotracheal tube to be inserted through the nasal cavity while the patient is conscious, minimally sedated, and breathing spontaneously [2]. This technique is typically employed in patients with anatomical abnormalities, cervical spine instability, or restricted mouth opening [1,2]. Possible complications of awake fiberoptic nasal intubation include oversedation, nasal bleeding, and complete obstruction of the airway [3].

To ensure airway protection and mechanical ventilation during a CABG procedure, endotracheal intubation is typically required [4]. However, for patients with a history of temporomandibular joint (TMJ) reconstruction, alteration in anatomy and restricted mouth opening present further challenges to airway management. In these circumstances, awake fiberoptic nasal intubation is required [5]. This report discusses the utilization of fiberoptic intubation in a patient who underwent a CABG post TMJ reconstruction.

This case was also presented as a medically challenging case at the 2023 Postgraduate Assembly in Anesthesiology Conference in New York City between December 8 and 11.

## Case presentation

**Figure 1:**
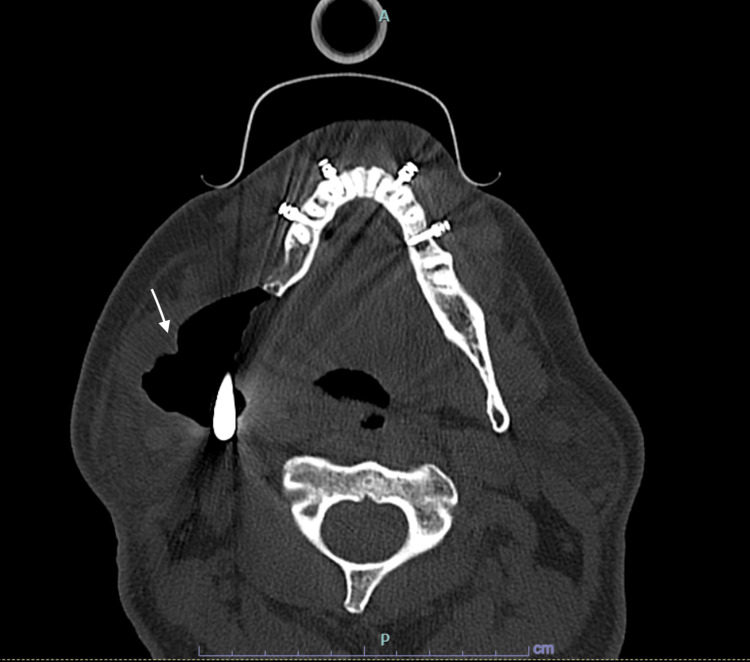
Axial CT scan of facial bones without contrast status post right mandible tumor resection and TMJ reconstruction Axial CT scan with bone window setting demonstrates an asymmetric enlargement of the right pterygoid and masseter muscles. A large amount of air is noted adjacent to the right mandibular prosthesis (arrow).

**Figure 2:**
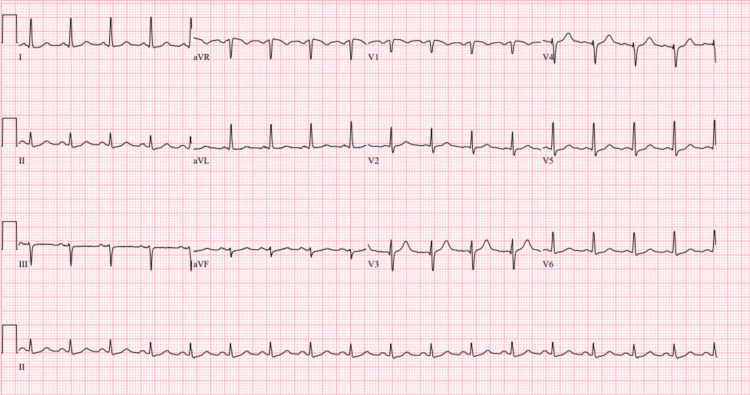
EKG on postoperative Day 1 of right mandible tumor resection and TMJ reconstruction surgery EKG shows ST depression in leads II, V2, V4-V6 TMJ: temporomandibular joint

A 56-year-old male, with a past medical history of benign ameloblastoma, gastroesophageal reflux disease, former smoker, 15-20 drinks/week, underwent elective surgery for right mandible tumor resection and reconstruction of his TMJ (Figure 1). A preoperative assessment revealed the patient was in no acute distress with a benign physical examination. Preoperative EKG showed normal sinus rhythm and rate without ischemic changes. The patient had a Revised Cardiac Risk Index Score of 0, indicating a low risk for major adverse cardiac events postoperatively. He was also classified as New York Heart Association (NYHA) Class I with good exercise tolerance and is functionally independent in all activities of daily living (ADLs). After an uneventful 11-hour intraoperative course, the patient complained of “chest tightness” in the postanesthesia care unit. He was tachycardic with a heart rate of 130 bpm and hypertensive with blood pressure of 153/98. Postoperative EKG showed diffuse ST depressions and elevated troponin at 5.15 ng/mL (Figure 2). After cardiology was consulted, he was prescribed metoprolol tartrate 50 mg twice daily, atorvastatin 80 mg, aspirin 81 mg, and placed on a heparin infusion. Subsequently, he was transported to the cath lab, where a coronary angiogram revealed severe left anterior descending and obtuse marginal disease. The cardiology team was unable to perform a percutaneous coronary intervention (PCI) during this time, with the patient suffering ventricular fibrillation (V-Fib) arrest and prompt return of spontaneous circulation after defibrillation. Coronary revascularization via coronary artery bypass graft (CABG) was recommended. Further imaging also revealed that he had an ejection fraction of 40% with a dilated right ventricle. This patient presented to the cardiac operating room (OR) one week later for a two-vessel CABG using left and right internal mammary arteries.

Upon arrival at the cardiac OR, our patient was pre-oxygenated at 15l/min via a non-rebreather mask. An infusion of dexmedetomidine at 1 mcg/kg/hr along with 1 mg of midazolam was given prior to the placement of a pre-induction arterial line and standard American Society of Anesthesiologists (ASA) monitors. The cardiac surgery team was asked to prep and drape the patient, who was in a semi-seated position, prior to induction. Next, his upper airway was topicalized with nebulized 4% lidocaine, and a lidocaine-coated nasal trumpet was inserted into the left nostril to check for patency. Midazolam and ketamine 10 mg IV were titrated to effect such that our patient was ventilating adequately, comfortable, and following commands. An oral 7.0 endotracheal tube, mounted on a slim Ambu® fiberoptic scope (Baltorpbakken, Denmark), was slowly advanced through the left nostril and past the vocal cords without trauma. Once the airway was secured, the patient was placed on sevoflurane, and paralysis was initiated. Transesophageal echo, which is routinely used in cardiac cases at our institution, was deferred given the lack of access to his esophagus. After an uneventful two-vessel CABG, our patient was transported to the ICU and extubated without incident on postoperative day 1 (POD1), with Anesthesia and ENT on standby for airway monitoring.

## Discussion

Difficult airways are an important consideration in the perioperative setting. Awake intubations are part of the difficult airway algorithm and represent a vital skill for the anesthesiologist. Awake intubations are recommended by the ASA practice guidelines whenever there is distorted airway anatomy [6]. Furthermore, acute or undiagnosed coronary artery disease in the perioperative setting may present more commonly than expected, with myocardial injury after noncardiac surgery occurring in roughly 20% of patients who have major inpatient surgery [7]. Our patient experienced a non-ST elevation myocardial infarction (NSTEMI) and V-fib arrest in the perioperative setting. Urgent coronary revascularization was critical; his only option was a CABG. Given his recent TMJ reconstruction and banding, our primary option involved an intranasal fiberoptic approach for intubation while maintaining adequate spontaneous ventilation. If this technique failed, our backup plan was to emergently cut through the elastic bands and attempt glidescope intubation, which would have further disrupted the anatomy of his reconstructed jaw. Lastly, the surgical team was asked to stand by for cricothyrotomy and/or emergent cardiopulmonary bypass.

Coronary artery disease places undue stress on the heart because of the imbalance between oxygen delivery and oxygen demand [8]. Factors that increase oxygen demand include increased heart rate, increased afterload, decreased hemoglobin, and a low fraction of inspired oxygen (FiO2) [9]. Conversely, we increase oxygen supply by increasing hemoglobin, FiO_2_, and of course, decreasing heart rate/afterload. Sympathetic surges, which can occur with anxiety, pain, and hypoxia, are routinely avoided in cardiac surgery, particularly during induction [10]. CABG surgery involving general anesthesia, cardiopulmonary bypass, and possibly prolonged ventilation can be anxiety-inducing to most patients [11]. Adding the need for an awake nasal fiberoptic intubation to that equation can be further traumatic on an emotional and physical level [12].

The attempt to circumvent this trauma, therefore, began before the patient even arrived in the operating room, when he first met his anesthesiology team on the floor. Our patient was educated on his condition; every step of the induction plan was explained thoroughly. The goal was to take away the element of surprise and to adequately prepare the patient with specific expectations. Since the patient was able to communicate by writing, all his concerns were adequately addressed. Upon arrival in the OR, each step of the process was explained again before being carried out, minimizing stress and anxiety along the way. These steps were taken to address his emotional well-being.

Next, we addressed his physical well-being. The details of induction and subsequent contingencies were discussed with and agreed upon by every member of the OR team before the patient arrived. All necessary equipment was double-checked; a second anesthesiologist was present in the room for assistance. The patient was preoxygenated with a non-rebreather mask at 15l/min to increase his FiO2, while his arterial line was placed. Midazolam was used in increments for anxiolysis. A dexmedetomidine infusion was started upon arrival in the OR to aid with sedation and to preserve respiratory function. Analgesia was achieved with ketamine, instead of opioids, to prevent respiratory depression. While it can be argued that ketamine might stimulate the sympathetic nervous system, small boluses are more likely to help with pain rather than cause large shifts in sympathetics [13]. Inhaled and topical lidocaine further decreased any noxious stimuli. Ultimately, the ETT was advanced and secured in the patient’s airway without causing any significant jumps in heart rate or afterload, while maintaining adequate spontaneous ventilation.

Following induction, his CABG was uneventful, and he was transferred to the ICU. Prior to extubation on POD1, we ensured that the patient was fully awake and alert, responding appropriately to commands and ventilating with adequate tidal volumes. A leak test was also performed to assess for airway edema. Anesthesia and ENT were present if the patient required reintubation. Once extubated, he recovered from both his surgeries without incident and was discharged from the hospital a few days later.

## Conclusions

This report narrates how to approach a difficult airway in a critically ill patient requiring a coronary artery bypass graft. It highlights some of the physiologic challenges we face when presented with these patients. Due to the patient’s recent history of temporomandibular joint reconstruction surgery, recent non-ST elevation myocardial infarction, reduced ejection fraction, and restricted oral access, it was necessary to use awake fiberoptic nasal intubation to achieve a secure airway and maintain spontaneous ventilation. Overall, clinical skills in combination with adequate preparation are essential when managing difficult airways. Adequately addressing a patient’s emotional needs may be just as important as addressing their physical needs during such tense situations. Clear communication and good bedside manners were imperative, as they reduced their psychological stress, which was a contributing factor to maintaining hemodynamic stability. The case report also highlights the importance of postoperative vigilance, as the immediate detection of the patient’s clinical symptoms and ischemic changes provided favorable patient recovery. Multidisciplinary communication between nurses, surgeons, and anesthesiologists is what ultimately guarantees successful patient outcomes.
